# A Longitudinal Assessment of Risk Factors and Chronic Diseases among Immigrant and Non-Immigrant Adults in Australia

**DOI:** 10.3390/ijerph18168621

**Published:** 2021-08-15

**Authors:** Mehwish Nisar, Tracy L. Kolbe-Alexander, Nicola W. Burton, Asaduzzaman Khan

**Affiliations:** 1 School of Health and Rehabilitation Sciences, The University of Queensland, Brisbane 4072, Australia; a.khan2@uq.edu.au; 2 School of Human Movement and Nutrition Sciences, The University of Queensland, Brisbane 4072, Australia; tracy.kolbe-alexander@usq.edu.au; 3 School of Health and Wellbeing, University of Southern Queensland, Ipswich 4350, Australia; 4 Division of Exercise Science and Sports Medicine, Department of Human Biology, Faculty of Health Sciences, University of Cape Town, Rondebosch, Cape Town 7700, South Africa; 5 School of Applied Psychology, Griffith University, Brisbane 4122, Australia; n.burton@griffith.edu.au

**Keywords:** immigrants, chronic disease, risk factors, Australia

## Abstract

This study aimed to investigate the prevalence and trajectories of chronic diseases and risk behaviors in immigrants from high-income countries (HIC), low–middle-income countries (LMIC), to Australian-born people. Data were used from five waves of the HABITAT (2007–2016) study—11,035 adults living in Brisbane, Australia. Chronic diseases included cancer, diabetes mellitus, coronary heart disease, and chronic obstructive pulmonary disease (COPD). Risk factors assessed were body mass index (BMI), insufficient physical activity, and cigarette smoking. Diabetes mellitus increased in all groups, with the highest increase of 33% in LMIC immigrants. The prevalence of cancers increased 19.6% in the Australian-born, 16.6% in HIC immigrants, and 5.1% in LMIC immigrants. The prevalence of asthma increased in HIC immigrants while decreased in the other two groups. Poisson regression showed that LMIC immigrants had 1.12 times higher rates of insufficient physical activity, 0.75 times lower rates of smoking, and 0.77 times lower rates of being overweight than the Australian-born population. HIC immigrants had 0.96 times lower rates of insufficient physical activity and 0.93 times lower rates of overweight than Australian-born. The findings of this study can inform better strategies to reduce health disparities by targeting high-risk cohorts.

## 1. Introduction

Chronic diseases are responsible for up to 70% of deaths worldwide [1]. According to the World Health Organization (WHO), the major chronic diseases are divided into four groups: heart disease, cancer, diabetes, and chronic obstructive pulmonary disease (COPD) [2]. These chronic diseases share common risk factors, including cigarette smoking, alcohol abuse, unhealthy diet, excess weight, and insufficient physical activity [3]. Tobacco smoking, excessive weight, and physical inactivity are the three main modifiable key risk factors that contribute substantially to the economic burden associated with chronic diseases. A modest annual 1% relative reduction in the prevalence of tobacco smoking, excess weight, and physical inactivity can reduce an annual $8.5 billion overall economic burden of chronic diseases [4].

Chronic diseases are responsible for 89% of all deaths in Australia [5]. One in two (50%) Australians have at least one of the following chronic diseases: arthritis, asthma, musculoskeletal problems, cancer, cardiovascular disease, COPD, diabetes, and mental health conditions [6]. In 2019, 11.6% of adults smoked daily while 15.6% of adults reported 11 or more alcohol drinks on 1 occasion at least once a year [7]. An estimated two in three adult Australians were overweight or obese and more than half of adults (55%) were not sufficiently active [7]. Tobacco smoking and alcohol use are responsible for 9% and 5.1% of the total burden of disease in the Australian population. Insufficient physical activity is associated with 10% and 20% of the disease burden of major chronic diseases [8].

Immigrants account for 29.7% of the Australian population [9]. Previous studies have shown that there is a large variation in the prevalence of chronic disease and modifiable risk factors in the Australian immigrant population [10]. Epidemiological data in Australia indicate that the disproportionate burden of disease among ethnic groups depends primarily on the country of origin, culture, language, and duration of stay in Australia. In particular, the different health behaviors and social needs of immigrants from low and high-income countries significantly impacted their overall well-being. The difference in the prevalence of chronic disease risk factors by country of birth was largely linked with their different financial circumstances. A large cross-sectional study of 264,102 Australians demonstrated that immigrants born in the high-income countries (HIC), had a higher mean chronic disease risk index than Australian-born participants, and immigrants from low–middle-income countries (LMIC), had a lower mean chronic disease risk index than Australian-born participants [11]. One study comparing ethnic groups reported that Sri Lankan/Bhutanese and Pacific Islander people were more likely to be hypertensive than other immigrant groups [12]. It was also reported that gestational diabetes is higher among pregnant women from South Asia (15.0%), Africa (9.4%), Vietnam (7.3%), than women born in Australia and New Zealand (4.3%) [13]. It is therefore important to differentiate between high and low/middle-income countries when studying immigrants’ health.

Most studies investigating the health of immigrants are cross-sectional, which only provides a snapshot in time of the differences between immigrants and Australian-born groups [11,12]. A small number of prospective studies have compared the incidence of chronic disease in immigrants and Australians [14]. However, none of these studies included a longitudinal assessment of the prevalence of chronic diseases and associated behavioral risk factors of immigrants from LMIC, HIC, and Australian-born people. This information is needed to identify the high-risk groups to be targeted for health care planning for chronic conditions. The aims of this study were, therefore, (a) to compare levels of chronic diseases and modifiable risk factors between Australian-born participants, low–middle-income countries (LMIC), and high-income countries (HIC) immigrant groups in a random sample of mid-aged and older adults living in Brisbane, Australia, and (b) to examine the trajectories of these diseases and risk factors over nine years. In this study, we focused on three modifiable risk factors: high body mass index, insufficient physical activity, and cigarette smoking; and four major chronic diseases, including cancer, diabetes mellitus, heart disease, and COPD (emphysema/chronic bronchitis and asthma). 

## 2. Methods

### 2.1. Setting and Recruitment

The data used in this analysis were from the HABITAT(How Areas in Brisbane Influence HealTh and AcTivity) study [15], which is a longitudinal, multilevel study that conducted a mail survey at five points approximately two years apart (2007, 2009, 2011, 2013, and 2016) in Brisbane. The 2016 census showed that 32.2% of Brisbane’s inhabitants were born overseas [16]. Migration streams in Brisbane are close to the national average (33.3% in 2016), which makes it a useful city for understanding immigrant health status in Australia [16]. The sampling and recruitment strategy for the HABITAT study has been detailed elsewhere [15]: a structured self-administered mail survey was first sent in May 2007 to 17,000 people (aged between 40 and 65 years), and 11,035 usable surveys were obtained, yielding a baseline response rate of 68.3% [15]. The corresponding response rates from eligible and contactable participants in 2009, 2011, 2013 and 2016 were 72.6%, 67.3%, 67.1% and 58.8%, respectively. The HABITAT study received ethical clearance from the Queensland University of Technology Human Research Ethics Committee (Ref. no. 3967H and 1300000161).

### 2.2. Measures

#### 2.2.1. Sociodemographic Measures

Individual-level sociodemographic measures included gender, age, educational qualifications, employment status, gross annual household income, and country of birth.

#### 2.2.2. Chronic Diseases

Health measures were derived from questionnaire items assessing global health status, and previously diagnosed chronic health diseases. Chronic diseases were assessed with the question, “Have you ever been told by a doctor or nurse that you have any of the long-term health diseases listed below? Please only include those diseases that have lasted, or are likely to last, for the last six months or more”. This list included asthma, any type of cancer, chronic bronchitis/emphysema, type 2 diabetes, heart/coronary disease. The answer required a yes/no response.

#### 2.2.3. Risk Factors

Physical activity (PA) was assessed using items from the Active Australia Survey to indicate time spent in the previous week [17]. Responses were quantified in MET-min/week as ([walking minutes × 3 METS] + [moderate minutes × 3 METS] + [vigorous minutes × 7.5 METS]), and categorized into one of two categories: ≥600 MET-Min/week for sufficient PA and <600 MET-min/week for insufficient PA [18]. Smoking status was assessed by asking “Which ONE of the following best describes your cigarette smoking?” Response options were, “I smoke daily”, “I don’t smoke now, but I used to”, “I smoke occasionally”, and “I have never smoked”. Body mass index (BMI) was calculated using the self-reported height and weight and divided into three categories: BMI < 25 = healthy weight, BMI 25–<30 = overweight, and BMI ≥ 30 = obese.

#### 2.2.4. Ethnicity

Participants self-reported their country of birth in response to the questions “Were you born in Australia?” and “If you were born in another country, where?” The World Bank country classification [19], by income group, was used to classify the country of birth of those participants who indicated they were not born in Australia. Because of known differentiated health effects of migration from LMIC to HIC, and HIC to HIC countries, the participants were divided into three groups based on their country of birth: (a) Australian-born, (b) immigrants from HIC and (c) immigrants from LMIC.

### 2.3. Data Analysis

Five waves of the HABITAT survey data were analyzed to determine the prevalence of self-reported chronic diseases and behavioral risk factors over 9 years by group country of birth: Australian-born, immigrants from high-income countries, and immigrants from low–middle-income countries. Poisson regression was used to estimate the prevalence ratios (PRs) because of its ability to estimate PR consistently and effectively in prospective studies. Poisson regression provides better analysis than logistic regression because of providing unbiased, more interpretable, and easier to communicate ratios [20]. To avoid overestimating the error of the estimated risk, a robust error variance procedure (sandwich estimation) was used (Zou, 2004). Preliminary bivariate analyses indicated that employment status, income, and education qualifications were not significantly associated with any of the diseases or risk factors under study. All regression models were adjusted for age, sex, education, and gross household income. All socio-demographic covariates analysed are listed in Table 1. We reported estimated PRs with 95% confidence intervals (CIs) at a significance level of *p* < 0.05. All analyses were conducted using Stata 14.0 SE.

## 3. Results

### 3.1. Participant Sociodemographic Characteristics

A summary of the sociodemographic variables of the HABITAT participants in wave 1 (baseline) (2007) is provided in Table 1. The average age was 51.04 (SD 7.06) years, and more than half of the respondents were women (55%). A quarter (25%) of the participants were born outside of Australia, with 16% from HICs and 9% from LMICs. Less than half of the participants (44%) considered their general health to be either “very good” or “excellent”, and 18% reported their health as fair to poor (Table 1).

#### 3.1.1. Chronic Diseases

**Figure 1:**
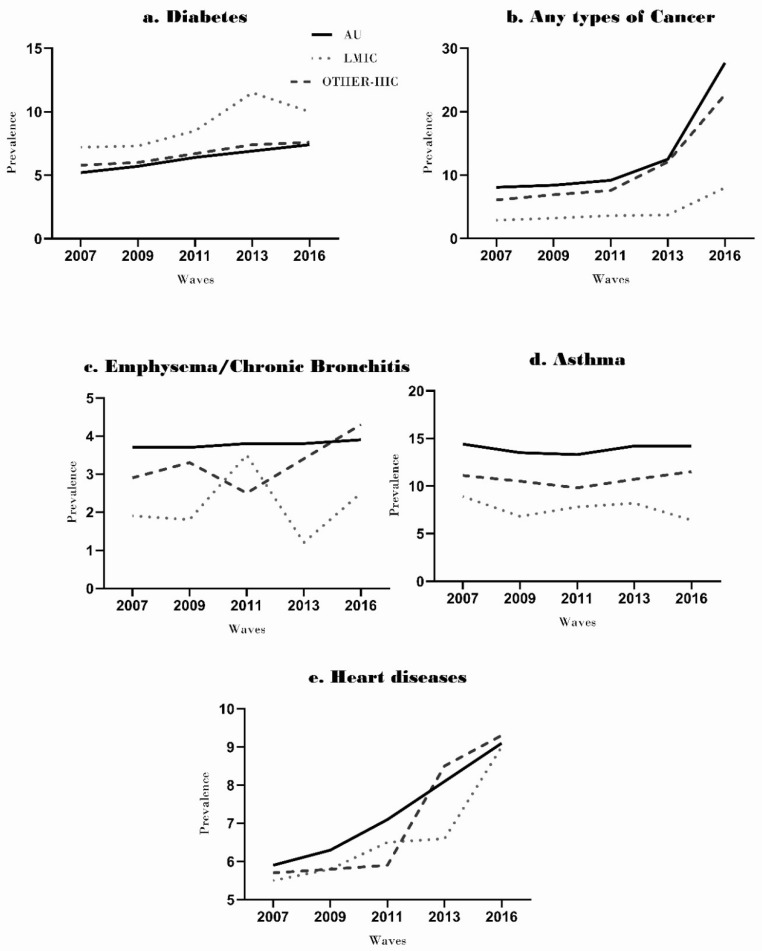
Prevalence of chronic diseases of immigrants (High-income countries and Low–middle-income countries) and Australian born people across five waves: 2007–2016 (**a**) prevalence of diabetes across five waves (**b**) prevalence of cancer across five waves (**c**) prevalence of emphysema/chronic bronchitis across five waves (**d**) prevalence of asthma across five waves (**e**) prevalence of heart diseases across five waves.

The prevalence of all chronic diseases (type 2 diabetes, heart/coronary disease, emphysema, and all types of cancer) increased over the nine years in all groups; the exception was asthma, which showed a decrease in LMIC and Australian-born groups. Diabetes mellitus increased in all groups, with the highest increase of 33% in LMIC immigrants (Figure 1a). The increased prevalence of any type of cancers was consistently higher among participants born in Australia (8.1% in 2007 to 27.7% in 2016) and immigrants from HICs (6.1% in 2007 to 22.7% in 2016) than those from LMICs (2.9% in 2007 to 8.0% in 2016). There was a decrease in the prevalence of asthma from 8.9% to 6.4% in LMIC immigrants, and from 14.4% to 14.2% in Australian-born, but an increase in HIC immigrants from 11.1% to 11.5% over nine years.

The overall prevalence of emphysema increased in all groups: LMIC immigrants (1.9% in 2007 to 2.5% in 2016), HIC immigrants (2.9% in 2007 to 4.3% in 2016) and Australian-born participants (3.7% in 2007 to 3.9% in 2016). However, the prevalence of emphysema showed a slight decline in some time points from previous values including, LMIC immigrants (wave 2 and wave 4), and HIC immigrants (wave 3), and Australian-born participants (wave 5) (Figure 1d). The prevalence of heart disease increased in all groups from 5.5% to 9.0% for LMIC immigrants, 5.7% to 9.3% for HIC immigrants, and 5.9% to 9.1% for Australian-born participants.

With the adjusted regression analysis, we found that LMIC immigrants had 1.02 times (95% CI 1.00–1.03) higher PRs of diabetes mellitus than Australian-born participants, while HIC immigrants showed similar rates as Australian-born participants. The PRs of emphysema 0.98 (95% CI 0.97–0.99), asthma 0.95 (95% CI 0.94–0.95) and cancers 0.93 (95% CI 0.92–0.95) were significantly lower in LMIC immigrants than Australian-born participants (*p* < 0.001). In HIC immigrants, the rates of emphysema 0.99 (95% CI 0.98–1.00) and asthma 0.98 (95% CI 0.96–0.99) were like the Australian-born participants (Table 2). Conversely, the rates of cancers 0.97 (95% CI 0.96–0.99) were significantly lower in HIC immigrants than the Australian-born participants (Table 2). The rates of heart diseases did not show any statistically significant difference between Australian-born, LMIC, and HIC immigrant groups.

#### 3.1.2. Risk Factors

**Figure 2:**
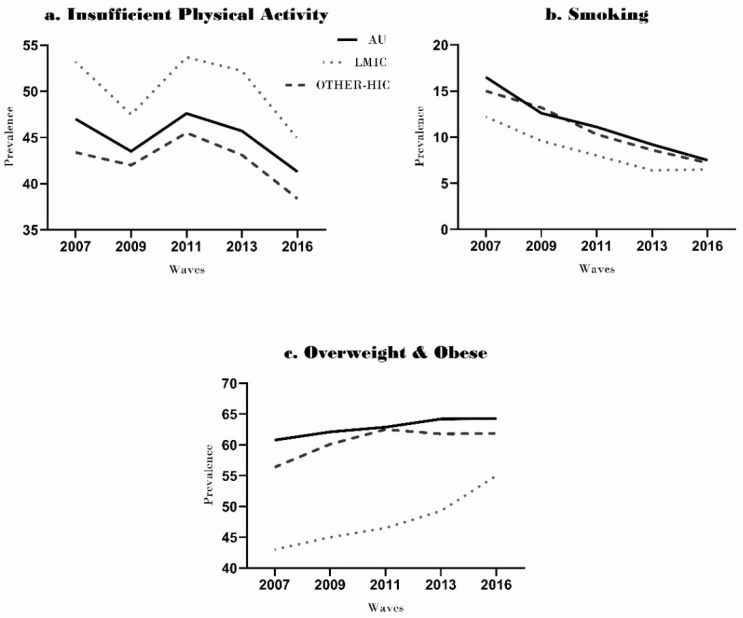
Prevalence of risk factors of immigrants (High-income countries and Low–middle-income countries) and Australian born people across five waves: 2007–2016. (**a**) prevalence of insufficient physical activity across five waves (**b**) prevalence of smoking across five waves (**c**) prevalence of overweight & obese across five waves.

Insufficient PA was consistently higher at each time point among LMIC immigrants (53.2% in 2007 to 44.9% in 2016) than HIC immigrants (43.4% in 2007 to 38.4% in 2016) and their Australian-born counterparts (47.1% in 2007 to 41.3% in 2016) (Figure 2a). The prevalence of overweight and obese peoples increased over nine years in LMIC immigrants (from 43.0% in 2007 to 55.0% in 2016), Australian-born (60.8% in 2007 to 64.3% in 2016) and HIC immigrant (56.4% in 2007 to 61.9% in 2016) (Figure 2b). The prevalence of smokers declined in all groups during the nine years. Smoking decreased more in HIC immigrants (14.9% in 2007 to 7.2% in 2016) than the Australian-born (16.5% in 2007 to 7.4% in 2016) and LMIC immigrants (12.2% in 2007 to 6.4% in 2016) (Figure 2c).

With Poisson regression, we found that the rates of insufficient PA were significantly higher in LMIC immigrants (1.12%, 95% CI 01.06–1.18; *p* < 0.001) than the Australian-born participants (Table 2). However, the rates of insufficient physical activity were similar in HIC immigrants and Australian-born people. The rates of smoking 0.75 (95% CI 0.63–0.91) and overweight participants 0.75 (95% CI 0.63–0.91) were significantly lower in LMIC immigrants than the Australian-born group. The PRs of overweight and obesity were also lower 0.93 (95% CI 0.90–0.97) in HIC immigrants than the Australian-born group, but the rates of smoking did not show any significant difference (*p* = 0.86) (Table 2).

## 4. Discussion

This study aimed to investigate the prevalence and trajectories of chronic diseases and risk factors in mid-aged Australian-born people and immigrants from LMICs and HICs. The prevalence of diabetes mellitus and insufficient physical activity was significantly higher in LMIC immigrants than Australian-born participants and HIC immigrants. Our results also showed that the prevalence of COPD, cancer, and smoking was lower in LMIC immigrants than in the other two groups.

This study found that the prevalence ratios of diabetes were 2% higher in LMIC immigrants than in the Australian-born population (Table 2). The higher rates of self-reported diabetes among LMIC immigrants are consistent with earlier studies [21]. It was reported that Indian migrants had an 11.1% prevalence of type 2 diabetes than the 2.9% self-reported prevalence among the general Australian population [22]. Transitioning and adjustment of cultures in a more developed geographical environment, unhealthy diet, lower economic status, and genetic makeup of immigrants are thought to be the major contributors to high diabetes rates among LMIC immigrants [21]. Our results also showed that the prevalence of diabetes increased among HIC immigrants (5.8% to 7.6%) and Australian-born participants (5.2% to 7.4%) from 2007 to 2016 (Figure 1a). This reflects the overall increase in the prevalence of diabetes in Australia. Obesity, low education level, smoking, and physical inactivity are related to increases in the incidence of diabetes among the Australian population [23].

Our research showed that the prevalence of cancer was lower in LMIC immigrants than in the other two groups. This difference may be because of discrepancies in diet, genetic makeup, and low cancer prevalence in the country of origin. Some studies have noted that compared with the host Australian population, migrants from LMIC had a lower risk of specific types of cancers (for example, breast, lung, and colorectal cancer) related to lifestyle, but a higher risk of other types of cancers (for example, cervical and liver cancer) related to infection [24]. The “healthy migrant effect” (health advantages of an immigrant at the time of migration [25]) could partly explain the low cancer prevalence in immigrants, but since prevalence remains low with a longer duration of stay, the influence of this effect is probably marginal [26].

We found that the prevalence of COPD was also lower in LMIC immigrants than HIC immigrants and the Australian-born. Another study reported a decline in the prevalence of COPD (from 7.3% to 7.1%) in LMIC immigrants between 2003 to 2009, with an increase from 12.7% to 14.3% for Australian-born [21]. Despite demonstrating a similar prevalence to other studies, our data also showed some non-uniform patterns of COPD prevalence in all groups (Figure 1c,d). This variation may be due to factors such as late diagnosis, decease participants, and a decline in smoking habits [21]. Our study results also showed a decline in the prevalence of smoking. Further research may elaborate on these differences between different immigrant groups and the Australian-born population.

The present study found no significant difference in the prevalence of chronic heart diseases between immigrants and non-immigrants. This is consistent with the results presented in the Australian health inequalities report [27] and Household Income and Labour Dynamics in Australia (HILDA) longitudinal survey [21]. The HILDA study indicated an increase in the prevalence of chronic heart diseases from 26.8% to 36.5% among immigrants, and from 24.1% to 36.4% among Australian-born over the nine years. Conversely, results from an analysis of the total population of New South Wales indicated significantly lower age-adjusted cardiac disease rates in the Australian-born population than migrants from the United Kingdom and Eire, Southern Europe, and Asia [28]. One likely explanation behind the similarity of our results among all groups may be the adaptation of host country environments and westernized diet over time [29]. A scoping review of the nutritional health of immigrants reviewed 49 articles and demonstrate that southeast Asians, Caribbeans, Africans, and Latinos are at high risk for nutrition-related chronic conditions including diabetes and heart disease [30].

Our investigation found higher levels of insufficient physical activity among LMIC immigrants, at each time point from HIC immigrants and Australian-born adults. Most of the previous studies with Australian immigrants also reported high levels of physical inactivity in immigrants [29]. The higher prevalence of insufficient physical activity among LMIC immigrants could be attributed to several factors, such as cultural beliefs, health attitudes, linguistic barriers, lack of motivation, financial problems, and lack of social support [31,32]. Another likely explanation for the difference in physical activity levels of LMIC immigrants and the Australian-born cohort might be the low levels of acculturation [33]. The acculturation hypothesis suggests that the level of physical activity among immigrants is strongly associated with the ability to speak the host language and to engage with the host culture [33,34]. This hypothesis might also explain why data in our study showed that immigrants from HICs had higher levels of physical activity than LMIC immigrants, as they may have better English language skills and be more acculturated to Australian society than those born in LMIC countries [34]. Further research is required to analyze the impact of different sociocultural barriers on the physical activity levels of LMIC immigrant groups.

All groups in this study showed a decrease in the prevalence of smoking over the nine years. This is similar to the decline in smoking prevalence in Australia, overall [35]. The main tobacco control measures to date have included an increase in mass media campaigns, increases in cigarette costs, and bans on smoking in public places [36]. The lower prevalence of smoking among LMIC immigrants than Australian-born participants in this study is consistent with most of the earlier studies [37]. Protective factors such as cultural and religious beliefs, social norms, and parental pressure against adopting smoking may contribute to the lower smoking rates among LMIC immigrants [37]. On the other hand, the lack of difference in tobacco smoking rates between immigrants from HIC and Australian-born participants may be due to similar exposure to culture, language, and socio-economic profile in their country of origin and Australia.

Our study observed exponential increases in the prevalence of overweight and obese people over the nine years among LMIC immigrants, as compared to HIC immigrants and Australian-born adults. Another study reported increased levels of excessive weight in LMIC immigrants, with a positive association with the duration of residence in Australia [12]. A possible explanation for the marked increase in weight among LMIC immigrant groups is the significant adoption in different ethnic groups of obesogenic behaviors (for example, consumption of more energy-dense and nutrient-poor foods, as well as less physical activity) following migration [38]. Despite the increase in the prevalence of overweight, this study also found that the prevalence ratios of overweight and obese in the LMIC group (0.77, 95% CI 0.72–0.82) and the HIC group (0.93, 95% CI 0.90–0.97) were still significantly lower than the Australian-born (*p* < 0.001). This is consistent with a previous Australian study which reported a significantly lower relative risk of obesity (0.81, 95% CI 0.79–0.83) in immigrants than the Australian-born population [11]. Another study also found that the first generation of immigrants had lower BMI than Australian-born, but had assimilated to the BMI of their host country in the second generation [39]. There is a need for research on the factors contributing to this accelerated increase in obesity in LMIC immigrants.

This study contributes substantially to the Australian immigrant health literature, particularly on risk factors and chronic diseases’ differences between immigrant and non-immigrant groups. This study used a large population-based sample over nine years, and this use of longitudinal analyses addressed some limitations of previous cross-sectional research (for example, time-bound and static representation) and provided data on trajectories. This study compared the Australian-born individuals with LMIC immigrants and HIC immigrants, which provides a better understanding of risk evaluation in different groups of immigrants.

However, this research has some limitations. These longitudinal data did not include information about dietary habits, which is one of the risk factors for most chronic diseases. The prevalence data were based on self-report, which is vulnerable to bias. However, the agreement between questionnaire data and medical records is robust for well-known chronic diseases that have clear diagnostic criteria and are easily communicated to the patients [40]. There were also no data available about the length of stay in Australia of immigrants, and therefore we could not analyze the association between health outcomes with the duration of residence. As the last wave was conducted in 2016, the results presented may not reflect the current conditions, which needs to be considered when interpreting the results.

## 5. Conclusions

This study found significant differences in the levels and trajectories of diabetes, cancers, COPD, insufficient physical activity, and overweight in LMIC and HIC immigrants and Australian-born adults. Data about chronic diseases and risk factors patterns in Australia’s immigrants are important to understand health gaps and the fundamental needs of different cultural groups. Health care planning targeting high-risk health behaviors (physical activity and weight management) is needed to ensure effective intervention and to maximize the impact of available human and financial resources. Further research is needed to better understand the underlying mechanisms associated with disparities in chronic diseases and their risk factors in different immigrant groups.

## Figures and Tables

**Table 1:** Sociodemographic and health characteristics of the analytical sample at baseline (*n* = 11,035), 2007.

*Characteristics*	*n*	%
Sex		
Male	4942	45.0
Female	6093	55.0
Age (years)		
42–44	2530	23.0
45–49	2382	21.0
50–54	2312	21.0
55–59	2080	19.0
60–65	1731	16.0
Country of birth *		
Australia	8245	75.0
High-income countries (HIC)	1704	16.0
Low–middle-income countries (LMIC)	1011	9.0
Employment status		
Full-time work	5846	53.0
Part-time work	1771	16.0
Home duties	683	6.0
Unemployed	160	1.0
Other	51	0.4
Highest educational qualification		
School only (up to 12 years)	4311	39.0
Certificate/diploma	3220	29.0
University degree	3457	32.0
Gross annual household income (AUD)		
$0–41599	2232	24.0
$41,600–72,799	2438	26.0
$72,800–129,000	2845	30.0
$130,000+	1889	20.0
General health status		
Excellent	1117	10.0
Very good	3670	34.0
Good	4194	38.0
Fair	1575	15.0
Poor	375	3.0
Body mass index (BMI)		
Healthy weight (<25)	4369	41.5
Overweight (25–<30)	3900	37.1
Obese (≥30)	2248	21.4

* HIC and LMICs are calculated by using the World Bank Atlas method (http://siteresources.worldbank.org/DATASTATISTICS/Resources/OGHIST.xls (accessed on 12 June 2021)).

**Table 2:** Prevalence ratios (PRs) ^¥^ of chronic diseases by country of birth and prevalence ratios (PRs) ^¥^ of risk factors by country of birth.

Country of Birth	Prevalence Ratio (PRs)	95% Confidence Interval	*p*-Value
**Chronic Diseases**			
Diabetes			
Australian-born participants	1.00 ^#^		
High-income countries	1.00	0.99–1.01	0.59
Low–middle-income countries	1.02	1.01–1.03	<0.00
COPD: Emphysema/chronic bronchitis			
Australian-born	1.00 ^#^		
High-income countries	0.99	0.98–1.00	0.07
Low–middle-income countries	0.98	0.97–0.99	<0.00
COPD: Asthma			
Australian-born participants	1.00 ^#^		
High-income countries	0.98	0.96–0.99	<0.00
Low–middle-income countries	0.95	0.94–0.95	<0.00
Heart diseases			
Australian-born participants	1.00 ^#^		
High-income countries	0.99	0.98–1.01	0.22
Low–middle-income countries	0.99	0.98–1.01	0.28
Cancer			
Australian-born participants	1.00 ^#^		
High-income countries	0.98	0.96–0.99	<0.00
Low–middle-income countries	0.93	0.92–0.95	<0.00
**Risk Behaviors**			
Cigarette Smoking			
Australian-born participants	1.00 ^#^		
High-income countries	0.99	0.87–1.12	0.86
Low–middle-income countries	0.75	0.63–0.91	<0.00
Insufficient Physical Activity			
Australian-born participants	1.00 ^#^		
High-income countries	0.96	0.91–0.99	0.04
Low–middle-income countries	1.12	1.06–1.18	<0.00
Overweight and Obese			
Australian-born participants	1.00 ^#^		
High-income countries	0.93	0.90–0.97	<0.00
Low–middle-income countries	0.77	0.72–0.82	<0.00

^#^ Reference category ^¥^ Adjusted for age, sex, education, and family income.

## Data Availability

The data that support the findings of this study are available on request from Gavin Turrell (gavin.turrell@rmit.edu.au).
